# *In vivo* vitamin D target genes interconnect key signaling pathways of innate immunity

**DOI:** 10.1371/journal.pone.0306426

**Published:** 2024-07-23

**Authors:** Julia Jaroslawska, Ranjini Ghosh Dastidar, Carsten Carlberg

**Affiliations:** 1 Institute of Animal Reproduction and Food Research, Polish Academy of Sciences, Olsztyn, Poland; 2 Institute of Biomedicine, School of Medicine, University of Eastern Finland, Kuopio, Finland; Tokushima University, JAPAN

## Abstract

The vitamin D_3_ metabolite 1,25-dihydroxyvitamin D_3_ (1,25(OH)_2_D_3_), its nuclear receptor VDR (vitamin D receptor) and hundreds of their target genes are not only key regulators of calcium homeostasis, but also important modulators of the immune system. Innate immune cells like monocytes use VDR for efficient differentiation and are very responsive to vitamin D. So far, most information on the gene regulatory function of vitamin D and its physiological impact had been obtained from *in vitro* studies using supraphysiological doses of 1,25(OH)_2_D_3_. Therefore, medical experiments like the study VitDHiD (NCT03537027), where 25 healthy individuals were supplemented once with a vitamin D_3_ bolus (80,000 IU), provide important insight into the response to vitamin D under *in vivo* conditions. In this study, we inspected 452 *in vivo* vitamin D target genes from peripheral blood mononuclear cells (PBMCs) detected in VitDHiD and found 61 of them involved in eight major KEGG (Kyoto Encyclopedia of Genes and Genomes) pathways of innate immunity. Under *in vivo* conditions in healthy individuals vitamin D either silences five pathways of innate immunity, stabilizes two and increases one, so that acute inflammation is suppressed and the release of cytokines is kept under control. A ranking of the 61 target genes by inducibility, basal expression and multiple involvements in the pathways highlighted the genes *NFKBIA* (NFκB inhibitor alpha), *NFKBIZ*, *FOSL2* (FOS like 2, AP1 transcription factor subunit), *JDP2* (Jun dimerization protein 2), *PIK3R1* (phosphoinositide-3-kinase regulatory subunit 1), *CLEC7A* (C-type lectin domain containing 7A), *DUSP6* (dual specificity phosphatase 6), *NCF2* (neutrophil cytosolic factor 2), *PLCB1* (phospholipase C beta 1), *PLCG2* and *TNFAIP3* (TNF alpha induced protein 3). In conclusion, vitamin D’s *in vivo* effect on innate immunity in healthy adults is mediated by the interconnection of the pathways of neutrophil extracellular trap formation, Toll-like receptor, chemokine and phagosome signaling, NOD-like receptor, C-type lectin receptor, apoptosis and interleukin 17 through a limited set of proteins encoded by key target genes.

## Introduction

The prohormone vitamin D_3_ has a central role in regulating calcium homeostasis, which is important for proper bone formation [1, 2]. Therefore, vitamin D deficiency can lead to bone malformation diseases like rickets in children and osteomalacia in adults [3]. The UV-B component of sunlight is needed for the cutaneous production of vitamin D_3_ from 7-dehydrocholesterol [4]. In humans, vitamin D_3_ is biologically inert and needs to be converted *via* 25-hydroxyvitamin D_3_ (25(OH)D_3_) to 1,25(OH)_2_D_3_ [5], which is the natural, high-affinity ligand of the nuclear receptor VDR [6, 7]. This transcription factor controls the expression of hundreds of vitamin D target genes [8]. In addition, the *VDR* gene is expressed in most human tissues and cell types. The observation that both moderate exposure to UV radiation as well as the supplementation with vitamin D_3_-rich cod liver oil is able to treat and prevent tuberculosis (an infectious disease caused by intracellular bacteria) [9, 10], was the first indication that vitamin D is able to modulate the actions of the immune system. Later on, it was noticed that a sufficient vitamin D status, *i*.*e*., a serum 25(OH)D_3_ concentration above 75 nM (30 ng/ml) [11], correlates with the reduced risk of autoimmune diseases, such as multiple sclerosis [12].

Vitamin D affects human immune cells, in particular those of the innate immune system, on multiple levels. For example, i) 1,25(OH)_2_D_3_ regulates the number of embryonic hematopoietic stem cells [13], ii) VDR is one of the key transcription factors in the differentiation process of myeloid progenitor cells into monocytes and granulocytes [14], which explains why these innate immune cells are most responsive to vitamin D [15], and iii) vitamin D modulates further differentiation of monocytes into dendritic cells and macrophages [16]. However, many molecular details, such as the identity of key vitamin D target genes being involved in these processes, are still largely lacking. An additional drawback is that in the past most of the studies were done using either model organisms like mice or cell lines, which were mostly of cancer origin. Furthermore, *in vitro* studies, such as with the monocytic cell line THP-1 or PBMCs isolated from human donors, used doses of 1,25(OH)_2_D_3_, such as 10 or even 100 nM, which are far higher than levels that can be reached under *in vivo* conditions [17, 18]. In order to address these problems, the vitamin D_3_ intervention trials VitDbol (NCT02063334, ClinicalTrials.gov) [19] and VitDHiD (NCT03537027) [20] had been designed as human *in vivo* studies, in which healthy individuals were supplemented once with a vitamin D_3_ bolus (80,000 IU). Both trials had their focus on primary molecular responses and therefore compared gene expression profiles from PBMCs isolated directly before vitamin D_3_ supplementation (d0) with those being obtained 24 h later (d1). This is a novel approach, since insight on the *in vivo* actions of vitamin D was previously primarily gained through the study of diseases related to vitamin D deficiency.

The study VitDHiD is of particular interest, since the transcriptome of 25 study participants had been investigated directly before and 24 h after supplementation [20]. This allowed the identification of 452 *in vivo* vitamin D target genes. A first analysis of these genes indicated that 88 of them are involved in 16 major signal transduction pathways listed in the database KEGG [21]. Interestingly, eight of these pathways were inhibited by vitamin D_3_ supplementation, while for the remaining eight pathways the contribution of vitamin D was approximately even concerning activation and inhibition, *i*.*e*., in the context of these signal transduction pathways vitamin D_3_ supplementation supported homeostasis.

In this study, we used the list of 452 *in vivo* vitamin D target genes from VitDHiD and identified 61 genes being involved in eight KEGG pathways of innate immunity. This provided further insight on the molecular details of the effects of vitamin D_3_ supplementation of healthy individuals.

## Materials and methods

### VitDHiD trial

Within the VitDHiD trial 25 healthy individuals (age 21–54, body mass index 21.4–25.6, basal 25(OH)D_3_ serum concentration 39–124.5 nM, S1 Table) had been supplemented with a single vitamin D_3_ bolus (80,000 IU). The bolus increased within one day the vitamin D status of the study participants to 73.8–160.3 nM, *i*.*e*., in average by some 35 nM (S1 Table). Blood was drawn directly before supplementation (d0) and 24 h later (d1) [20]. Compliance to the supplementation was measured through an increase in vitamin D_3_ serum level by an average of 91.2 ng/ml, while serum calcium levels did not change significantly in any of the participants (S1 Table). All research was performed in accordance with relevant guidelines and regulations. The ethics committee of the Northern Savo Hospital District had approved the study protocol (#515/2018). All participants gave written informed consent to participate in the study.

### Identification and characterization of *in vivo* vitamin D target genes

In the VitDHiD trial, PBMCs were isolated within one hour after blood draw from 20 ml of peripheral blood in Vacutainer CPT Cell Preparation Tubes with sodium citrate (Becton Dickinson) according to the manufacturer’s instructions (S1 Fig). Total RNA was extracted using the High Pure RNA Isolation kit (Roche) following the manufacturer’s protocol. RNA quality was assessed on an Agilent Bioanalyzer and library preparation was performed after rRNA depletion applying kits and protocols from New England Biolabs. RNA-seq libraries were sequenced at 75 bp read length on a NextSeq500 system (Illumina) using standard protocols at the Gene Core of the EMBL (Heidelberg, Germany). Fastq files of the raw data can be found at Gene Expression Omnibus (GEO, www.ncbi.nlm.nih.gov/geo) with accession numbers GSE260981. Differential gene expression was computed using *EdgeR* [22], which implements a negative binomial test over the reads in the two conditions (d1/d0), with standard parameters, a FDR (false discovery rate) cutoff of 0.05 and logCPM greater than 10.

### Relating vitamin D target genes to pathways of innate immunity

From the list of 452 *in vivo* vitamin D target genes identified in the VitDHiD trial [20], 61 were found to participate in eight major pathways of innate immunity listed in the database KEGG [23] (www.genome.jp/kegg). These 61 *in vivo* vitamin D target genes were compared with target gene lists from PBMCs [17, 20, 24, 25] and THP-1 human monocytes [26] and 49 were confirmed as known targets (S2 Table). All genes were inspected manually for their function, such as type of encoded protein and its primary biological function, with the help of databases like Human Protein Atlas (www.proteinatlas.org) and GeneCards (www.genecards.org). Networks of interacting genes were plotted according to rules of KEGG signaling pathway diagrams. In each pathway a set of genes (nodes) was connected, in order to reflect the functional relationships between them. Arrows connecting one node with another indicate positive downstream effects, while lines ending with a shorter perpendicular line display inhibitory effects. The nodes were colored based on the logFC (fold change) in gene expression between d1 and d0 (downregulation in red, upregulation in green). White nodes indicate genes expressed in PBMCs but not being significantly regulated by vitamin D_3_ bolus supplementation. Asterisks at the nodes imply that the respective genes are not listed in KEGG but encode for a close functional partner or a member of the same family. The arrow next to the gene name in each colored node indicates the overall net effect of vitamin D_3_ supplementation on the expression of the respective gene. Thus, not only the vitamin D-induced direction of regulation of gene expression (up or down) but also the function of a particular gene (inhibitor or activator) in the signaling pathway is considered. An arrow pointing up indicates that the vitamin D-induced regulation of expression of the gene contributes to signal amplification in a particular signaling pathway, while an arrow pointing down demonstrates signal inhibition. When arrows pointing in opposite directions at a given node, vitamin D-induced gene regulation has a mixed effect on the signal transduction flow. The net overall regulatory effect of vitamin D exerted on a pathway was calculated by summing the inducibility (median logFC) of all target genes implicated in that pathway (Table 1). After applying arbitrarily established threshold value ranges, if the resulting value was below 0.1, we considered that vitamin D had an inhibitory effect on pathway regulation, values above 0.1 had a stimulatory effect. Intermediate values indicate no regulation, which implies stabilization of the pathway rather than any polarization. All innate immune response signaling pathways listed under the category of “Cellular Processes” and “Organismal System” in KEGG were examined in detail, but only the eight pathways containing at least nine vitamin D target genes are presented.

**Table 1:** The net regulatory effect of vitamin D_3_ supplementation on eight pathways of innate immunity.

Pathway	Number of vitamin D target genes	Sum of median logFC	Net effect on pathway regulation
Toll-like receptor (TLR)	17	-1,712	down
C-type lectin receptor (CLR)	12	-1,611	down
NOD-like receptor (NRL)	12	-0,972	down
APOPTOSIS	10	-0,330	down
Neutrophil extracellular trap (NET)	19	-0,122	down
Phagosome	14	-0,037	none
Interleukin 17 (IL17)	9	-0,021	none
Chemokine	16	0,436	up

### Characterization of VDR-binding enhancers

The genomic regions of key *in vivo* vitamin D target genes were investigated for VDR binding enhancer and TSS (transcription start site) regions by using epigenome-wide data from THP-1 cells, which had been stimulated for 24 h with 10 nM 1,25(OH)_2_D_3_ or solvent (0.1% EtOH). These were ChIP-seq (chromatin immunoprecipitation sequencing) data of i) the histone marker of active TSS regions, H3K4me3 [27], ii) the histone marker of active chromatin, H3K27ac [27] and iii) VDR [28] as well as FAIRE-seq (formaldehyde-assisted identification of regulatory elements followed by sequencing) data [26]. With help of the IGV-browser [29] these data sets were displayed for the genomic regions of the vitamin D target genes and VDR enhancers showing responsiveness to 1,25(OH)_2_D_3_ were marked. One Mb of the genomic region up- and downstream of the target gene’s TSS were screened but only essential regions are shown.

## Results

### *In vivo* vitamin D target genes are primarily involved in signal transduction

In the VitDHiD trial [20], changes of gene expression 24 h after a supplementation with a vitamin D_3_ bolus (80,000 IU) were measured for 25 healthy individuals. Transcriptome-wide analysis indicated 452 *in vivo* vitamin D target genes in PBMCs (FDR < 0.05 and logCPM > 10), 61 of which were found to encode for proteins participating in eight major pathways of innate immunity outlined in the database KEGG (S2 Table). Forty-nine of these 61 genes have already been described in human PBMCs [17, 20, 24, 25], THP-1 cells [26] or in other cellular systems, such as *GADD45A* in the context of prostate and colon cancer cells [30, 31], to respond significantly to 1,25(OH)_2_D_3_. These known vitamin D target genes encode for the adaptor proteins DDX3X (DEAD-box helicase 3 X-linked), GADD45A (growth arrest and DNA damage inducible alpha), GADD45B, GNG11 (G protein subunit gamma 11), KSR1 (kinase suppressor of ras 1), NCF2, NFKBIA, NFKBIZ, NLRP12 (NLR family pyrin domain containing 12), PELI1 (pellino E3 ubiquitin protein ligase family member 1), PELI2, PIK3R1, S100A8 (S100 calcium binding protein A8), S100A9 and TNFAIP3, the cell adhesion protein THBS1 (thrombospondin 1), the water channel AQP9 (aquaporin 9), the enzymes ADCY9 (adenylate cyclase 9), CTSZ (cathepsin Z), DUSP6, FGR (FGR proto-oncogene, Src family tyrosine kinase), MEFV (MEFV innate immunity regulator, pyrin), NAMPT (nicotinamide phosphoribosyl transferase) and PADI2 (peptidyl arginine deiminase 2), PADI4, PLCB1, RIPK2 (receptor interacting serine/threonine kinase 2) and THEM4 (thioesterase superfamily member 4), the chemokine CXCL5 (C-X-C motif chemokine ligand 5), the receptors C5AR1 (complement C5a receptor 1), CCR7 (C-C motif chemokine receptor 7), CD14 (CD14 molecule), CSF2RB (colony stimulating factor 2 receptor subunit beta), CLEC4D, CLEC7D, CXCR4 (C-X-C motif chemokine receptor 4), CX3CR1 (C-X3-C motif chemokine receptor 1), FCGR2A (Fc gamma receptor IIa), FPR1 (formyl peptide receptor 1), HLA-DQB1 (major histocompatibility complex, class II, DQ beta 1), ITGB3 (integrin subunit beta 3), ITGB5 and TREM1 (triggering receptor expressed on myeloid cells 1), the transcription factors BCL3 (BCL3 transcription coactivator), FOSL2, IRF5 (interferon regulatory factor 5), JPD2 and RELB (RELB proto-oncogene, NFκB subunit) as well as the transporter ATP6V0B (ATPase H^+^ transporting V0 subunit b).

The remaining 12 genes are novel *in vivo* vitamin D targets in immune cells (S2 Table). They encode for the adaptor proteins ARRB2 (arrestin beta 2) and NLRC3 (NLR family CARD domain containing 3), the chloride channel CLCN3 (chloride voltage-gated channel 3), the chromatin protein H2AC6 (H2A clustered histone 6), the enzymes MAP3K20 (mitogen-activated protein kinase kinase kinase 20), PLCE1 and PLCG2, the receptor ITGA2B, the structural proteins TUBB4B (tubulin beta 4B class IVb), TUB4A and TUBB1 as well as the transporter SLC25A4 (solute carrier family 25 member 4).

In summary, only 12 of the 61 investigated genes have not been described previously in human immune cells as targets of vitamin D. Known and novel vitamin D target genes encode proteins of signal transduction cascades, such as ligands, receptors, adaptor proteins, regulatory enzymes and transcription factors.

### *In vivo* vitamin D target genes involved in KEGG pathways of innate immunity

The involvement of 61 *in vivo* vitamin D target genes (S2 Table) in eight pathways of innate immunity was analyzed next. The pathways were drawn in the style of KEGG and nodes representing vitamin D target genes were colored. The number of genes being reliably related to signaling pathways is still limited and so far KEGG has assigned only some 20% of all human genes to one or multiple pathways [23]. We extended the pathway nodes by close functional partners and/or members of the same protein family. In summary, 19 vitamin D target genes were identified in the pathway NET formation (S2 Fig), 17 in TLR signaling (S3A Fig), 16 in chemokine signaling (S4A Fig), 14 in the phagosome pathway (S5 Fig), each 12 in the NRL pathway (S3B Fig) and the CRL pathway (S6 Fig), 10 in the apoptosis pathway (S7 Fig) as well as 9 in IL17 signaling (S4B Fig).

The majority of the 61 *in vivo* vitamin D target genes (42, representing 68.9% of all) were downregulated after vitamin D_3_ bolus supplementation, while only 19 were upregulated in their expression. In contrast, most of the proteins encoded by the 61 genes (50, *i*.*e*., 82.0%) have an activating role, whereas only eight genes encoding the transcription factor BCL3, the enzymes DUSP6 and THEM4 as well as the adaptor proteins ARRB2, NFKBIA, NFKBIZ, NLRC3 and TNFAIP3 act as inhibitors. Interestingly, the enzyme MEFV and the adaptor protein NLRP12 in the NLR pathway and the enzyme CTSZ in the apoptosis pathway have both inhibitory as well as activating functions (S3B and S7 Figs). The number of upregulated genes varies between the eight investigated pathways and ranges from each one of the vitamin D targets of CRL and IL17 signaling to each six of the chemokine and phagosome pathways. This represented 8.3–42.9% of the vitamin D targets of the pathways.

NETs are extracellular traps composed of chromatin and antimicrobial proteins, that are released by neutrophils and macrophages to capture exogenous pathogens and prevent their spread. 24 h after vitamin D_3_ bolus supplementation the expression of genes encoding several cell surface receptors mediating phagocytosis, such as CLEC7A, FPR1, FCGR2A and C5AR1, was significantly decreased in immune cells (S2 Fig). Moreover, genes coding for the two peptidyl arginine deaminases PAD2 and PAD4, which catalyze reactions of histone citrullination modifications required for extrusion of necroptotic NETs, were also downregulated. On the other hand, genes encoding for members of integrin family, ITGA2B and ITGB3, being involved in platelet-induced NET formation, were upregulated. Thus, in healthy individuals vitamin D_3_ supplementation affects NET formation at multiple levels. However, vitamin D triggers rather the genes involved in the lytic component of NET release than in vital NETosis. The net physiological effect of vitamin D on the NETs formation pathway is inhibitory (the sum of median logFC values for 19 genes involved in a pathway equals -0.122, Table 1).

Elevation of the vitamin D status in the blood of healthy individuals affected the expression of genes whose products signal primarily through a MyD88 (myeloid differentiation factor 88)-dependent component of the TLR signaling pathway (S3A Fig). In the absence of TLR ligand engagement, vitamin D_3_ supplementation decreases the expression of the TLR4 coreceptor CD14 as well as of a receptor amplifying the inflammatory response triggered by TLR, TREM1. Furthermore, vitamin D downregulates the expression of adaptor proteins PELI1 and PELI2 and the enzyme RIPK2 along with components of the AP1 (activating protein 1) transcription factor complex, JDP1 and FOSL2. An important exception to the repressive effect of vitamin D on TLR signaling are two inhibitors of NFκB (NFKBIA and NFKBIZ), the downregulation of which favors the translocation of the transcription factor NFκB from the cytoplasm to the nucleus and the activation of multiple proinflammatory target genes. In contrast, the recruitment of intracellular TLRs and downstream signaling through the adaptor protein TRIF (TIR-domain containing adapter inducing interferon β) appears to be unaffected by vitamin D_3_ supplementation. In net effect, vitamin D suppressed the TLR signaling pathway (the sum of median logFC values for 17 genes involved in a pathway equals -1.712, Table 1).

The effects of vitamin D on the chemokine signaling pathway include altered expression of genes encoding the chemokine ligand CXCL5, the chemokine receptors CXCR4, CCR7 and CX3CR1 as well as several proteins involved in chemokine signaling, all of which may result in changes in cytokine production and cell polarization (S4A Fig). By influencing the expression of genes encoding proteins whose signaling leads to calcium mobilization and diacylglycerol (DAG) production as well as by downregulating NCF2, which is a cytosolic subunit of a NADPH oxidase, vitamin D can modulate the level of intracellular ROS (reactive oxygen species) production. In net effect, signaling of the chemokine pathway as well as the immunomodulatory processes regulated by this pathway seem to be moderately activated after vitamin D_3_ supplementation (the sum of median logFC values for 16 genes involved in a pathway equals 0.436, Table 1).

Vitamin D_3_ supplementation significantly changed the expression level of genes encoding several phagocytosis-promoting receptors. Most of these target genes were downregulated (S5 Fig). On the other hand, genes encoding tubulin structural proteins (TUBA4A, TUBB4B and TUBB1), which are involved in microtubule dynamics, facilitating maturation, movement and fusion of phagosomes with other organelles of the endocytic pathway, were upregulated by vitamin D. In healthy individuals, vitamin D_3_ supplementation regulates the transcription of genes involved in few phases of phagosome formation, and the effect of this regulation is to maintain the balance of these processes (the sum of median logFC values for 14 genes involved in a pathway equals -0.037, Table 1).

NLRs are important intracellular pattern recognition receptors that mediate immune recognition by forming multi-protein complexes called inflammasomes. Vitamin D_3_ bolus supplementation did not significantly affect NLRs expression but changed the expression of several genes encoding proteins involved in the downstream signaling, such as the MEFV enzyme and the adaptor protein NLRP12 (S3B Fig). In a pathogen-specific manner both proteins function either as activators of the inflammasome triggering the release of the proinflammatory cytokines IL1β and IL18 or as negative regulators. Beyond the inflammasome, supplementation with vitamin D_3_ altered the expression of genes encoding proteins acting upstream (RIPK2 and TNFAIP3) or as part of inflammatory pathways (DUSP6, JDP1, FOSL2, NFKBIA and NFKBIZ). The net effect of vitamin D on the NLR pathway is inhibitory (the sum of median logFC values for 12 genes involved in a pathway equals -0.972, Table 1).

Vitamin D reduced the expression of pattern recognition receptors of the family, such as CLEC7A and CLEC4D, which recognize fungi-derived ß-glucan and the mycobacterial surface lipid TDM (trehalose dimycolate), respectively (S6 Fig). The expression of many components of the intracellular signaling cascade, including the adaptor proteins PIK3R1, KSR1, NFKBIA and NFKBIZ, the enzymes PLCG2 and DUSP6 as well as the transcription factors BCL3, JDP2, FOSL2 and RELB also changed significantly. The net effect of vitamin D on the CLR pathway is inhibitory (the sum of median logFC values for 12 genes involved in a pathway equals -1.611, Table 1).

The onset of apoptosis is controlled by many interconnected processes. Most of the vitamin D target genes involved in apoptosis mediate signaling through the extrinsic (membrane receptor-mediated) pathway, while only the *CTSZ* gene is involved in intrinsic (mitochondria-mediated) pathway (S7 Fig). Overall, in immune cells of healthy individuals, vitamin D_3_ supplementation leads to a predominance of apoptotic over prosurvival signaling (the sum of median logFC values for 10 genes involved in a pathway equals -0.330, Table 1).

Vitamin D_3_ supplementation did not affect any of the receptors specific for the IL17 signaling pathway but altered the expression of the several elements of NFκB and MAPKs downstream signaling as well as of inflammatory chemokines (CXCL5) and antimicrobial peptides (S100A8 and S100A9) (S4B Fig). Among all IL17 family members, vitamin D_3_-induced changes in gene expression are most closely associated with cell subsets targeted by IL17A and IL17F. The latter are primarily involved in host protection against extracellular pathogens, but also in promoting inflammatory pathology in autoimmune diseases. The sum of median logFC values for 9 genes involved in IL17 signaling equals -0.021 (Table 1), which means that the pathway remains stable after vitamin D_3_ supplementation.

Taken together, the majority of the eight investigated pathways of innate immunity are downregulated. This suggests that under *in vivo* conditions vitamin D inhibits innate immunity in healthy individuals. However, only in the case of chemokine production, vitamin D_3_ supplementation seems to have stimulating effect. For phagosome and IL17 signaling, the change in the expression level of target genes exerted by vitamin D is neither stimulatory nor inhibitory, *i*.*e*., in the context of these signaling pathways, vitamin D_3_ supplementation rather supported homeostasis.

### Ranking of *in vivo* vitamin D target genes

**Fig 1:**
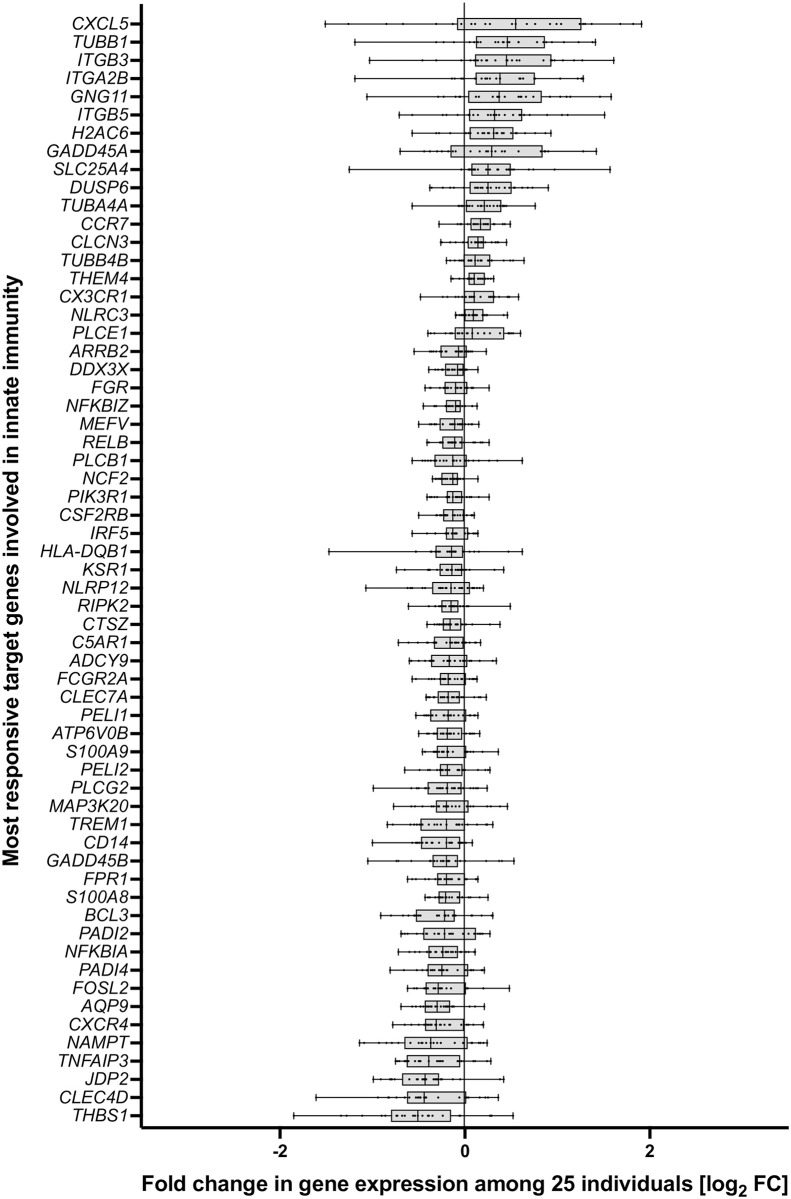
Range of inducibility of *in vivo* vitamin D target genes involved in pathways of innate immunity. Box plots indicate the variation in the inducibility of gene expression (logFC) of the 25 individuals participating in VitDHiD.

The 61 *in vivo* vitamin D target genes involved innate immunity can be classified by their inducibility (Fig 1). Based on the median inducibility, the 10 most upregulated genes are *CXCL5*, *TUBB1*, *ITGB3*, *ITGA2B*, *GNG11*, *ITGB5*, *H2AC6*, *GADD45A*, *SLC25A4* and *DUSP6*, while the 10 most downregulated genes are *THBS1*, *CLEC4D*, *JDP2*, *TNFAIP3*, *NAMPT*, *CXCR4*, *AQP9*, *FOSL2*, *PADI4* and *NFKBIA*. The remaining 41 genes are in average between 1.16-fold upregulated and 1.17-fold downregulated. However, there is a large interindividual variation in the regulation of each of the genes.

**Fig 2:**
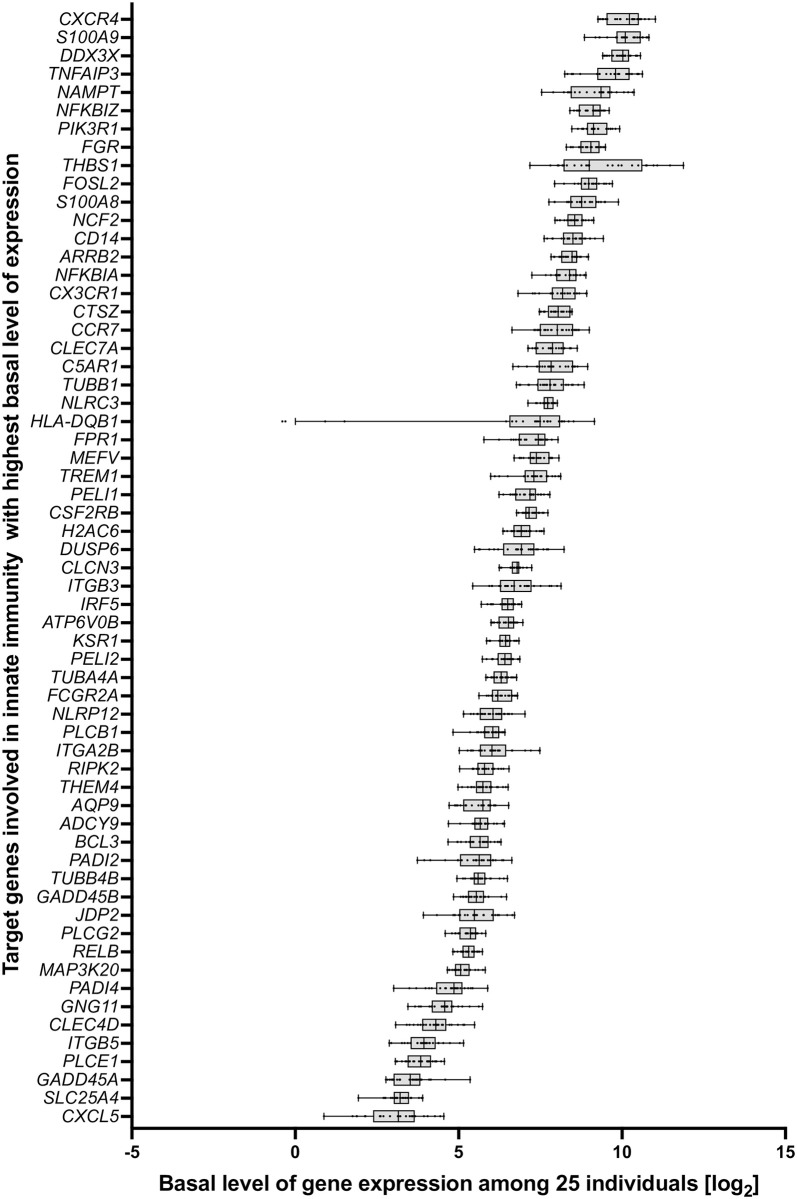
Range of basal expression of *in vivo* vitamin D target genes involved in pathways of innate immunity. Box plots indicate the variation of basal gene expression (log scale) of the 25 individuals participating in VitDHiD. The genes are sorted by median average expression.

Similarly, the 61 genes can be ranked by their median basal expression, which is highest for *CXCR4*, *S100A9*, *DDX3X*, *TNFAIP3* and *NAMPT* and some 100-fold lower for the weakest expressed genes *CXCL5*, *SLC25A4*, *GADD45A*, *PLCE1* and *ITGB5* (Fig 2). Also concerning basal expression there is large interindividual variation, which is highest for *HLA-DQB1*, *THBS1*, *CXCL5*, *PADI2* and *PADI4* und lowest for *NLRC3*, *RELB*, *TUBA4A*, *CSF2RB* and *ATP6V0B* (S2 Table). A third way of ranking the 61 *in vivo* vitamin D target genes is how often they occur in the eight investigated pathways (S2 Table). This is each 7-times for *NFKBIA* and *NFKBIZ*, each 5-times for *FOSL2*, *JPD2* and *PIK3R1*, each 3-times for *CLEC7A*, *DUSP6*, *NCF2*, *PLCB1*, *PLCG2* and *TNFAIP3* as well as twice for *ARRB2*, *CD14*, *CLEC4D*, *CXCL5*, *FCGR2A*, *ITGA2B*, *ITGB3*, *MAP3K20*, *NLRC3*, *PLCE1*, *RIPK2* and *TUBA4A*. The remaining 38 genes occur in only one of the eight pathways of innate immunity.

**Fig 3:**
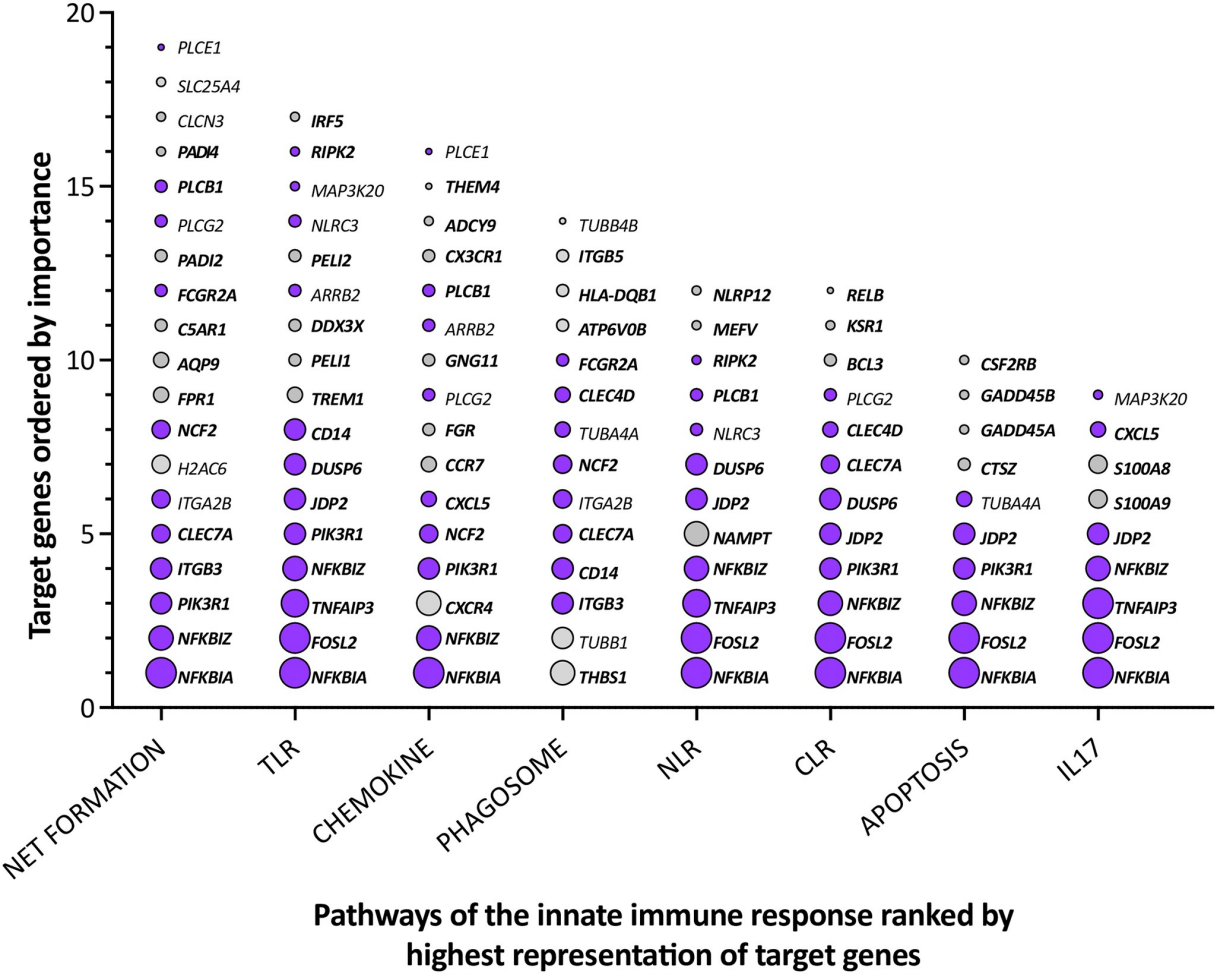
Ranking of *in vivo* vitamin D target genes involved in innate immunity. The 9–19 vitamin D target genes involved in the eight major pathways of innate immunity are ranked by inducibility, basal expression and involvement in multiple pathways (S2 Table). The sizes of the circles are proportional to the ranking position. Purple color indicates involvement of a gene in two or more pathways. Gene names in bold signify already known vitamin D targets. In summary, seven of eight pathways have the *NFKBIA* gene as top ranking candidate followed by the genes *FOSL2*, *TNFAIP3* and *NFKBIZ*. Only the phagosome pathway has with *THBS1* and *TUBB1* an individual set of top ranking genes.

Overall, the joint assessment of all three classification options places the gene *NFKBIA* first ranking before the genes *FOSL2* and *TNFAIP3* (S2 Table). When analyzing the joint ranking for each of the eight pathways separately, the top 3 for NET formation are *NFKBIA*, *NFKBIZ* and *PIK3R1*, for TLR signaling *NFKBIA*, *FOSL2* and *TNFAIP3*, for chemokine signaling *NFKBIA*, *NFKBIZ* and *CXCR4*, for the phagosome pathway *THBS1*, *TUBB1* and *ITGB3*, for NRL signaling *NFKBIA*, *FOSL2* and *NFKBIZ*, for CLR signaling *NFKBIA*, *FOSL2* and *NFKBIZ*, for the apoptosis pathway *NFKBIA*, *FOSL2* and *NFKBIZ* and for IL17 signaling *NFKBIA*, *FOSL2* and *TNFAIP3* (Fig 3).

### Interconnection of pathways of innate immunity by *in vivo* vitamin D target genes

**Fig 4:**
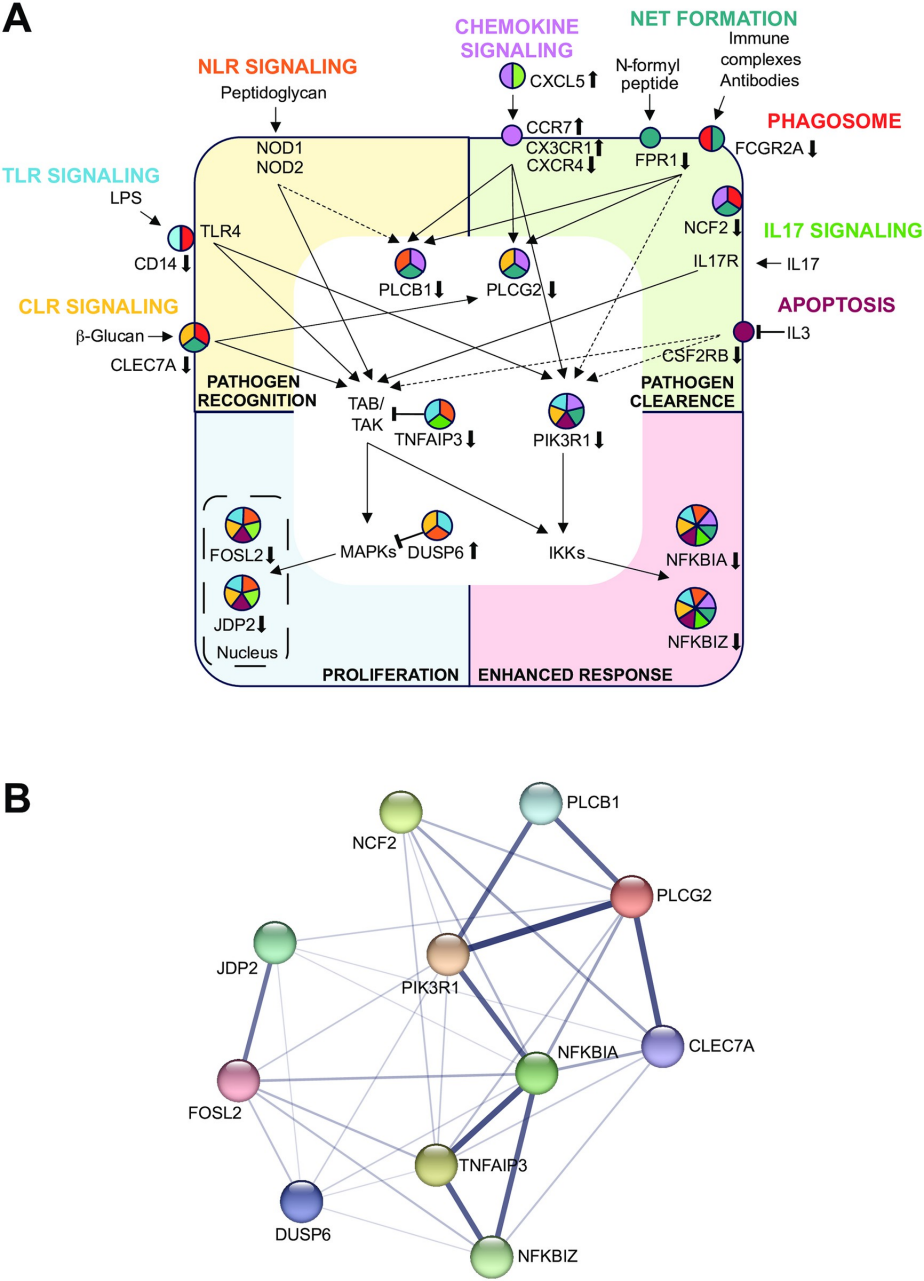
Interconnection of key *in vivo* vitamin D target genes in innate immunity. A schematic model of a cell illustrates the location and connection of key *in vivo* vitamin D target genes (circles) (**A**). Arrows next to the gene names indicate up- and downregulation. The size of the circles is proportional to the number of pathways (differently color coded), in which the genes are involved. Four major vitamin D-triggered physiological processes, “pathogen recognition”, “pathogen clearance”, “proliferation” and “enhanced response”, are distinguished. A protein-protein interaction network of the 11 key genes appearing 3 or more times in the eight investigated KEGG pathways was visualized by STRING version 12.0 (http://string-db.org) (**B**). The nodes indicate proteins and the edges integrate both functional and physical types of protein associations. The thickness of the lines between nodes indicates the strength of data support for each interaction.

The eight major pathways of innate immunity modulate the physiological processes “pathogen recognition”, “pathogen clearance”, “proliferation” and “enhanced response” (Fig 4A). All pathways start with membrane receptors that are activated by stimuli, such as beta-glucan (mimicking fungi; CRL signaling *via* CLEC7A), lipopolysaccharides (LPS, mimicking extracellular bacteria; TLR signaling *via* CD14 and TLR4), peptidoglycans (representing extracellular matrix; NLR signaling *via* NOD (nucleotide-binding oligomerization domain-containing protein) 1 and NOD2), chemokines like CXCL5 (chemokine signaling *via* chemokine receptors like CCR7, CX3CR1and CXCR4), N-formyl peptide (stimulating NET formation *via* FPR1), antibodies and immune complexes stimulating phagocytosis (phagosome pathway *via* Fc receptors like FCGR2A and cytosolic adaptor proteins like NCF2), IL17 (activating the IL17 pathway) and withdrawal of IL3 (stimulating apoptosis *via* CSF2RB). Some of these pathways use either the adaptor proteins TNFAIP3 and PIK3R1 or the enzymes PLCB1, PLCG2 or DUSP6 as mediators, in order to modulate the activity of the components of the proliferation-stimulating transcription factor complex AP1, JDP2 and FOSL2, or inhibitors of the proinflammatory transcription factor NFκB, NFKBIA and NFKBIZ. The interconnection of the proteins encoded by the 11 key *in vivo* vitamin D target genes is visualized with the help of the database STRING [32] (Fig 4B). This indicates that there is good experimental evidence that the proteins NFKBIA, NFKBIZ, TNFAIP3, PIK3R1, PLCB1, PLCG2 and CLEC7A functionally interact, while DUSP6 and NCF2 are only loosely connected to this network. Furthermore, FOSL2 and JDP2 are strongly connected to each other (forming an AP1 complex) but are only loosely connected to the other proteins.

A screening for VDR-bound enhancer and TSS regions using published ChIP-seq and FAIRE-seq datasets from THP-1 cells identified for all 11 key genes one or two VDR-binding enhancers in a distance ranking from very close or identical to the TSS (*NFKBIA*, *NCF2*, *JDP2*, *DUSP6*, *PIK3R1* and *NFKBIZ*) and up to 1270 kb apart from the TSS (S8 Fig). Accordingly, all 11 genes seem to be directly regulated by ligand-triggered VDR, but their enhancers can be distinguished into strong VDR binders (*NFKBIA*, *FOSL2*, *NCF2*, *JDP2* and *PLCG2*, S8A Fig) and weaker VDR binders (*DUSP6*, *TNFAIP3*, *PIK3R1*, *PLCB1*, *CLEC7A* and *NFKBIZ*, S8B Fig).

Taken together, the *in vivo* vitamin D target genes *NFKBIA*, *NFKBIZ*, *FOSL2*, *JDP2*, *PIK3R1*, *CLEC7A*, *DUSP6*, *NCF2*, *PLCB1*, *PLCG2* and *TNFAIP3* are key nodes of major pathways of innate immunity, most of which form a functional network. Moreover, all 11 key vitamin D target genes have a VDR-bound enhancer in reasonable vicinity of their TSS region.

## Discussion

This study analyzed the molecular consequences of vitamin D_3_ supplementation on the transcriptome of 25 healthy individuals participating in the VitDHiD trial [20]. Since the study protocol of VitDHiD (NCT03537027) only allowed the collection of PBMCs, we focused on effects on the immune system. Assessing human vitamin D signaling through medical experiments like in VitDHiD, which uses vitamin D_3_ bolus supplementation [24], is a new approach identifying vitamin D target genes. The VitDHiD study was designed to investigate transcriptome-wide changes that were induced by vitamin D_3_ bolus supplementation already with 24 h. In this way, a short-term *in vivo* effect of vitamin D can be analyzed. This is in contrast to other studies, such as that of Hossein-nezhad *et al*. [33], which investigated gene expression changes of vitamin D_3_ supplementation over a period of 8 weeks.

Since vitamin D is known to affect more efficiently innate than adaptive immunity [14–16], we had main attention on pathways of innate immunity listed in KEGG. We took advantage of a list of 452 *in vivo* vitamin D target genes of VitDHiD and found in total 61 genes involved at least once in the eight major pathways of innate immunity. Most of these target genes (80%) had already been reported by *in vitro* experiments. Thus, the design of our study as a medical experiment fulfilled our expectation that this *in vivo* setup can resemble classical *in vitro* experiments using 1,25(OH)_2_D_3_ as a stimulus. Interestingly, some of the 61 genes, such as *CD14*, are downregulated *in vivo*, while their expression was found to be upregulated *in vitro*. The downregulation of CD14 and the whole TLR pathway by vitamin D_3_ supplementation makes sense for healthy individuals, since vitamin D prevents an overreaction of the innate immune system in contact with microbes [34]. However, we do not fully understand the mechanistic basis of this oppositive direction of response, but assume that it is related to the indirect effects of vitamin D *via* its influence on signal transduction pathways, which are regulated environmental signals differing between the *in vivo* and *in vitro* setting, such as cytokines, growth factors and peptide hormones [17].

The very most of the 61 genes have an activating function, but since the majority of genes are downregulated by vitamin D_3_ supplementation, the overall effect of vitamin D on five out of eight pathways of innate immunity is their downregulation. The only exception is chemokine signaling, for which six nodes function as activators of the pathway and six other nodes as their inhibitors. When we sum up the median values of inducibility of all 16 target genes expression (logFC) of the 25 individuals participating in VitDHiD, we see that in the case of chemokine production, vitamin D is stimulating these processes. However, also for the pathways of IL17 signaling and phagocytosis, the prevalence of inhibitory signals over activating ones is rather small. This indicates that for these pathways the action of vitamin D is not strongly polarized and maintains them in a state close to equilibrium. Thus, the overall effect of raising the vitamin D status is the inhibition or balancing of innate immune responses. This observation makes sense, since VitDHiD was studying healthy individuals [20] that within the 24 h, in which the vitamin D_3_ bolus was applied and blood samples were provided, had no obvious immune challenge. Therefore, there was no need that vitamin D activates innate immunity but rather represses it, in order to prevent molecular stress and accidental overreactions. This may be signified best at the example of TLR signaling [35], in which 17 nodes are responsive to vitamin D but 13 of them have a repressive effect. In addition, the pathways of other pattern-recognition receptors, CLR and NRL, are also downregulated. Since a major consequence of the activation of these pathways is the induction of acute inflammation, in healthy individuals vitamin D_3_ supplementation seems to represses inflammation. This fits with our previous observation that vitamin D counteracts in PBMCs LPS-induced inflammation [25].

An apparent shortcut for the manual inspection of signaling pathways, as performed in this study, would be the use of web-based tools like EnrichR [36]. However, EnrichR provides inaccurate results with a rather short list of only 61 genes. Moreover, EnrichR depends on the content of databases like KEGG, *i*.*e*., genes that are not intensively studied are underrepresented. Moreover, the gene list enrichment analysis tool lacks the context of the expression changes, *i*.*e*., the direction and magnitude of the regulation of the enriched processes cannot be determined solely based on these results. Thus, to our experience, tools like EnrichR are useful for getting some first general hypotheses on the physiological consequences of stimulating vitamin D endocrinology, but are not suited for more detailed information on the response of signaling pathways.

The detailed inspection of vitamin D-triggered pathways of innate immunity identified nodes, such as the proteins NFKBIA and NFKBIZ, that are used by up to seven of the eight investigated pathways. In total, 23 of the 61 investigated *in vivo* vitamin D target genes occur in at least two pathways, *i*.*e*., *via* these genes vitamin D seems to have larger impact on innate immunity than through the remaining 38 genes that are used only in one of the eight pathways.

The function of vitamin D to influence innate immunity has been demonstrated in a great number of experimental models, but the molecular mechanisms involved in these processes are not yet entirely clear. As we show here, the mechanism of action of vitamin D in immune cells should be viewed as a coordinated change in the expression of target genes, which creates a network of interactions linking various immune processes together, rather than the regulation of signaling *via* linear pathways. Based on frequency of occurrence of 61 *in vivo* vitamin D target genes in the studied pathways, we identified 11 hub genes, whose transcriptional products connect eight major innate immunity pathways. *PLCG2* is a novel vitamin D target in human immune cells, while the 10 other genes had already been known as vitamin D targets from *in vitro* experiments. The *PLCG2* gene encodes for a member of transmembrane signaling phospholipases C (PLC) that catalyze the conversion of 1-phosphatidyl-1D-myo-inositol 4,5-bisphosphate to 1D-myo-inositol 1,4,5-trisphosphate (IP3) and DAG. This reaction uses calcium as a second messenger and mediates intracellular signaling downstream of growth factor and immune system receptors. Interestingly, 1,25(OH)_2_D_3_ has been shown to upregulate the expression of another PLCG isoform, PLCG1, in rat colonocytes [37], human keratinocytes [38] and human Jurkat T cells [39] as well as PLCB1, both *in vivo* (mouse costochondral cartilage [40] and rat growth plate chondrocytes [41]) as well as *in vitro* (human keratinocytes [42]).

In this study, we reported 12 novel *in vivo* vitamin D target genes with potential immunomodulatory function. Importantly, the identification of these target genes occurred in immune cells of healthy individuals in the absence of factors that stimulate the immune response and at physiological serum vitamin D levels, which are much lower than the pharmacological doses of 1,25(OH)_2_D_3_ in *in vitro* experiments. The 61 *in vivo* vitamin D_3_ target genes show a wide range both in their basal expression and inducibility. The highly expressed target genes *TNFAIP3*, *NFKBIZ* and *PIK3R1* are downregulated in response to vitamin D_3_ supplementation, while low expressed genes like *CXCL5* are upregulated. In this way, vitamin D reduces the expression range of this gene set. Thus, extremes in gene expressions are prevented and the cellular systems move towards homeostasis. However, the wide range in the inducibility of the investigated 61 vitamin D target genes suggests that there are interindividual differences in the responsiveness to vitamin D. This supports our concept of a vitamin D response index, which segregates individuals into high, mid and low responders to vitamin D [43].

In conclusion, based on the manual inspection of the role of 61 *in vivo* vitamin D target genes within eight pathways of innate immunity, we observed an overall inhibition or stabilization of these pathways. This suggests that in healthy individuals a sufficient vitamin D status leads to the silencing of innate immunity, such as a suppression of inflammation. The proposed model for vitamin D action in immune cells is an active suppression or maintenance in balance the innate immune signaling through coordinated regulation of several interaction points to ensure an accurate, non-exaggerated molecular response. Key vitamin D targets in this process are the genes *NFKBIA*, *NFKBIZ*, *FOSL2*, *JDP2*, *PIK3R1*, *CLEC7A*, *DUSP6*, *NCF2*, *PLCB1*, *PLCG2* and *TNFAIP3*. Keeping innate immunity processes repressed/stable seems to be the most adequate response in the absence of immune challenge.

## Supporting information

S1 Fig Visual illustration of the workflow of this study. (PDF)

S2 Fig NET formation pathway. Representation of the NET formation pathway following the design of KEGG. The given percentage reflects the number of *in vivo* vitamin D target genes, whose direction of regulation will contribute to pathway inhibition. Upregulated vitamin D targets are labeled green and downregulated red. Color intensity is proportional to logFC of gene expression between d1 and d0. Functionally similar proteins that are not indicated in KEGG are marked by an asterisk.
(PDF)

S3 Fig TLR and NRL pathways. Representation of the pathways TLR (**A**) and NLR (**B**) following the design of KEGG. The given percentages reflect the number of *in vivo* vitamin D target genes, whose direction of regulation will contribute to pathway inhibition. Upregulated vitamin D targets are labeled green and downregulated red. Color intensity is proportional to logFC of gene expression between d1 and d0. Functionally similar proteins that are not indicated in KEGG are marked by an asterisk.
(PDF)

S4 Fig Chemokine and IL17 signaling pathways. Representation of the pathways chemokine (**A**) and IL17 (**B**) signaling following the design of KEGG. The given percentages reflect the number of *in vivo* vitamin D target genes, whose direction of regulation will contribute to pathway inhibition. Upregulated vitamin D targets are labeled green and downregulated red. Color intensity is proportional to logFC of gene expression between d1 and d0. Functionally similar proteins that are not indicated in KEGG are marked by an asterisk.
(PDF)

S5 Fig Phagosome pathway. Representation of the phagosome pathway following the design of KEGG. The given percentages reflect the number of *in vivo* vitamin D target genes, whose direction of regulation will contribute to pathway inhibition. Upregulated vitamin D targets are labeled green and downregulated red. Color intensity is proportional to logFC of gene expression between d1 and d0. Functionally similar proteins that are not indicated in KEGG are marked by an asterisk.
(PDF)

S6 Fig CRL pathway. Representation of the CRL pathway following the design of KEGG. The given percentages reflect the number of *in vivo* vitamin D target genes, whose direction of regulation will contribute to pathway inhibition. Upregulated vitamin D targets are labeled green and downregulated red. Color intensity is proportional to logFC of gene expression between d1 and d0. Functionally similar proteins that are not indicated in KEGG are marked by an asterisk.
(PDF)

S7 Fig Apoptosis pathway. Representation of the apoptosis pathway following the design of KEGG. The given percentages reflect the number of *in vivo* vitamin D target genes, whose direction of regulation will contribute to pathway inhibition. Upregulated vitamin D targets are labeled green and downregulated red. Color intensity is proportional to logFC of gene expression between d1 and d0. Functionally similar proteins that are not indicated in KEGG are marked by an asterisk.
(PDF)

S8 Fig VDR-binding enhancers in the genomic regions of vitamin D target genes. The IGV browser was used to visualize ChIP-seq results for H3K4me3 (purple) [27], H3K27ac (green) [27] and VDR (red) [28] as well as FAIRE-seq data (turquois) [26] obtained in THP-1 cells that had been treated for 24 h with solvent (EtOH) or 1,25(OH)_2_D_3_ (1,25D). The target genes are classified based on strong (**A**) and weaker (**B**) VDR binding to their enhancers and TSS regions (shaded in grey). The peak tracks display merged data from the three biological repeats. Gene structures are shown in blue and vitamin D target genes are highlighted in red. The genomic regions 1 Mb up- and downstream of each gene’s TSS were inspected but only the areas relevant for 1,25(OH)_2_D_3_-dependent regulation are displayed.
(ZIP)

S1 Table VitDHiD study participants. Age, gender, BMI and serum levels of vitamin D_3_, Ca and 25(OH)D_3_ as well as percentage of monocytes in PBMCs at d0 and d1 are indicated.
(XLSX)

S2 Table List of 61 *in vivo* vitamin D target genes being involved in eight major pathways of innate immunity. The 61 selected vitamin D target genes are characterized. All data related to the analysis presented in this study are contained in this table. Fastq files of the raw data can be found at Gene Expression Omnibus (GEO, www.ncbi.nlm.nih.gov/geo) with accession numbers GSE260981.
(XLSX)
