# Supplementary figures and images for: In vivo vitamin D target genes interconnect key signaling pathways of innate immunity
Source: PLoS One. 2024 Jul 23;19(7):e0306426. doi: 10.1371/journal.pone.0306426 (PMC11265685; doi:10.1371/journal.pone.0306426)

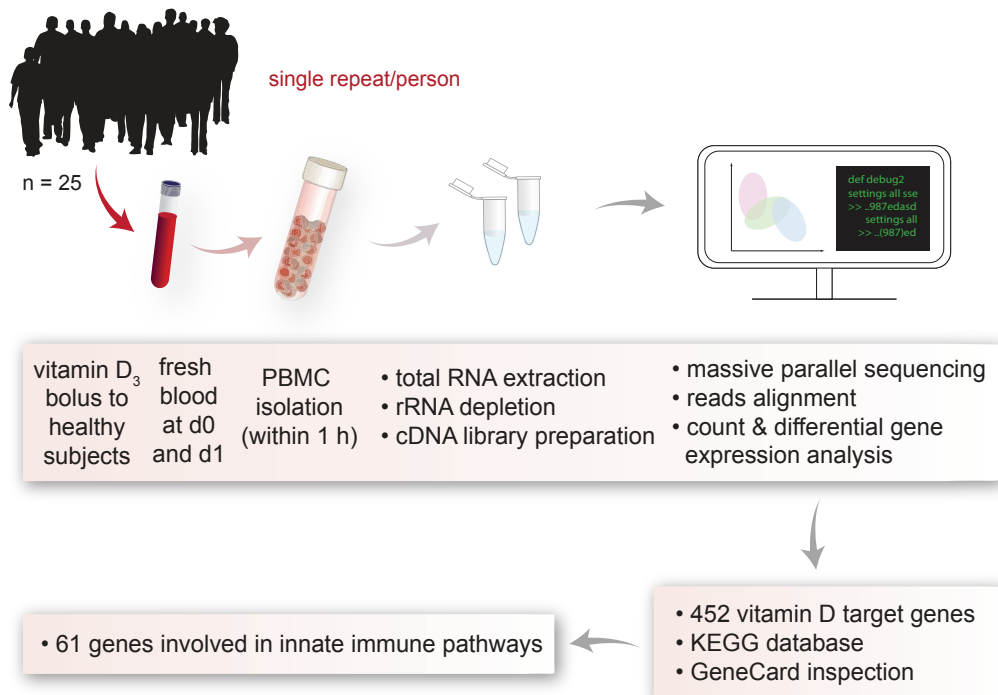

Supplement: S1 Fig — (PDF) [file pone.0306426.s001.pdf]

# NET FORMATION (57.9%)

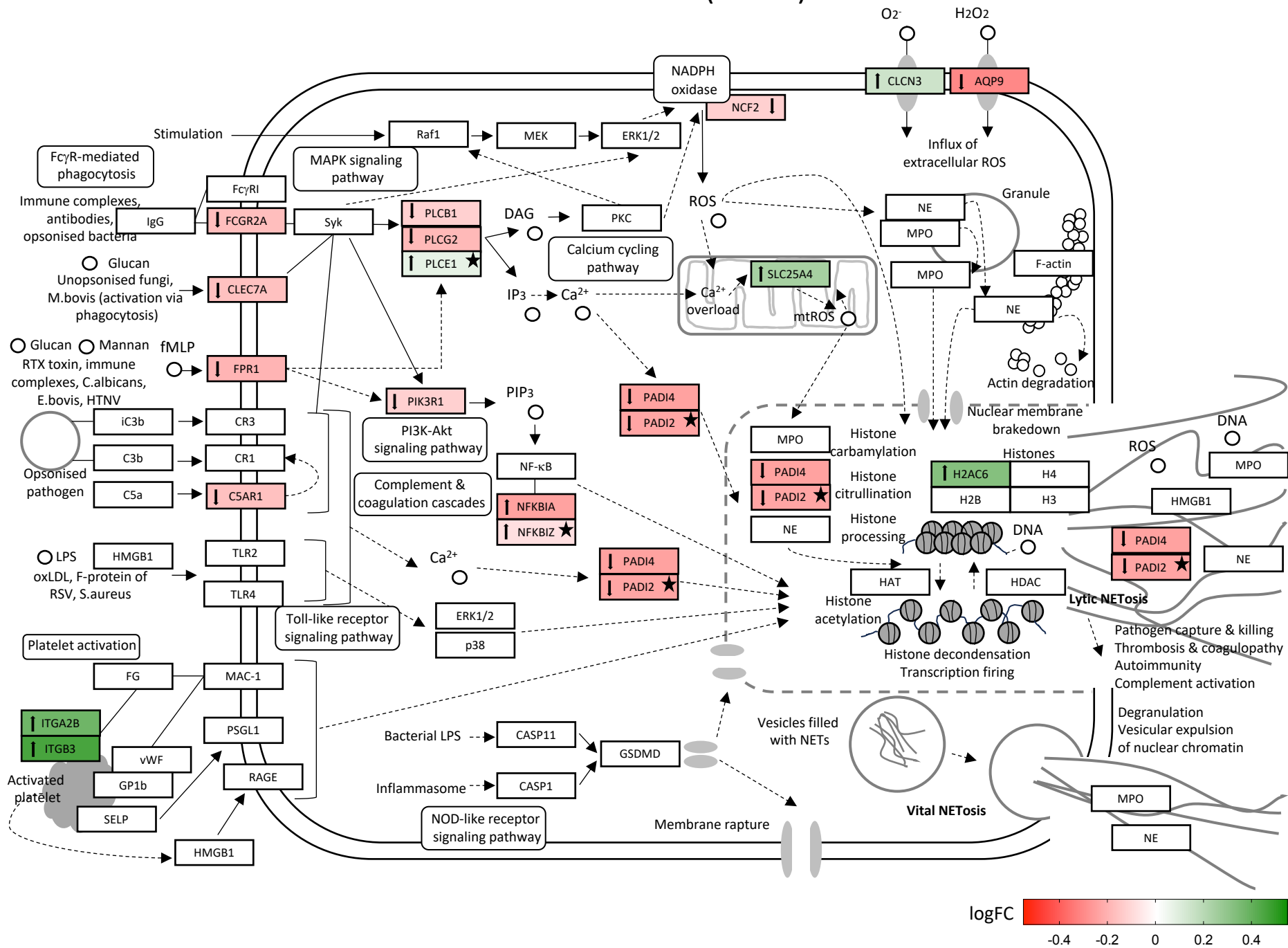

Supplement: S2 Fig — Representation of the NET formation pathway following the design of KEGG. The given percentage reflects the number of in vivo vitamin D target genes, whose direction of regulation will contribute to pathway inhibition. Upregulated vitamin D targets are labeled green and downregulated red. Color intensity is proportional to logFC of gene expression between d1 and d0. Functionally similar proteins that are not indicated in KEGG are marked by an asterisk. (PDF) [file pone.0306426.s002.pdf]

B

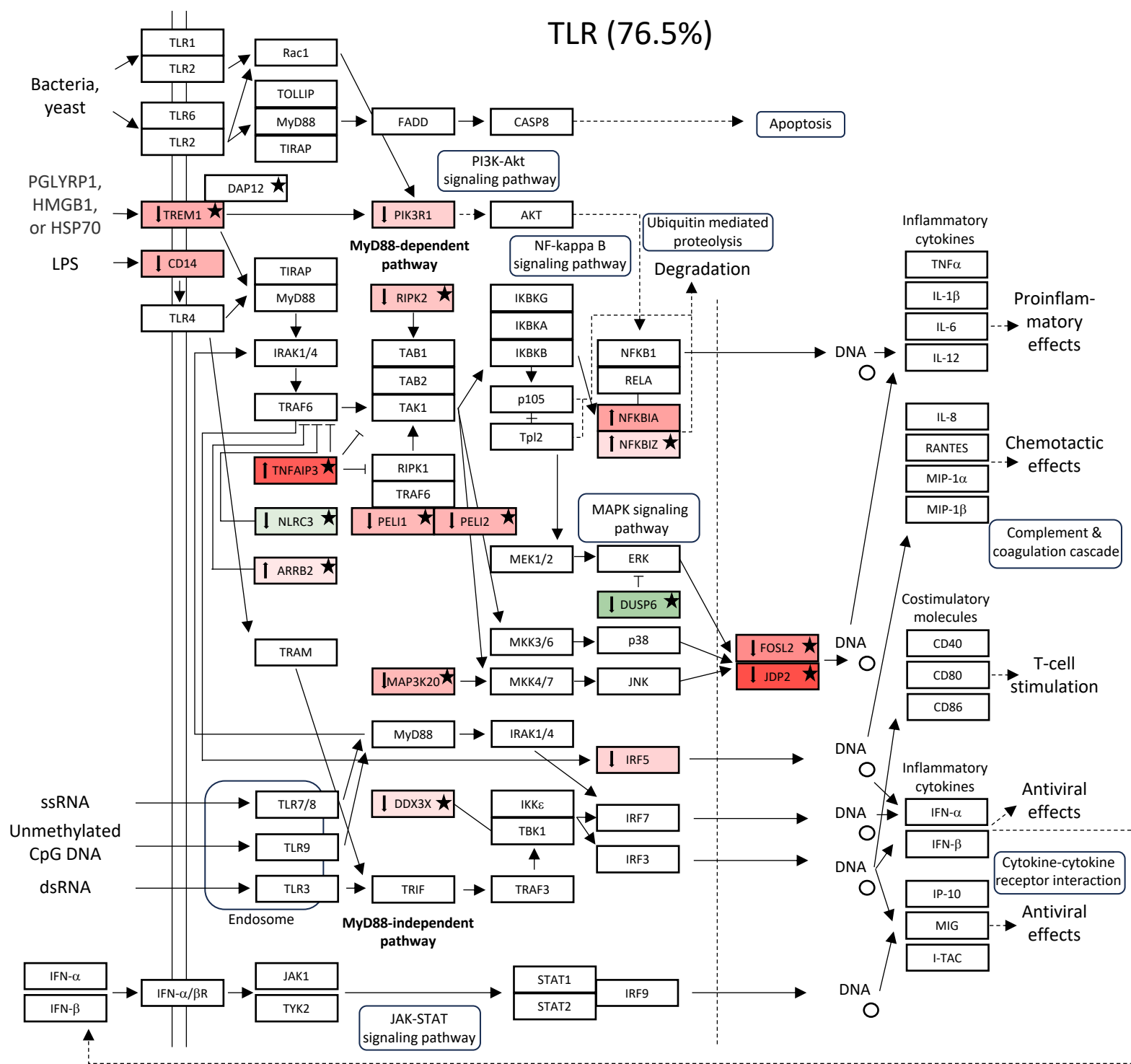

B

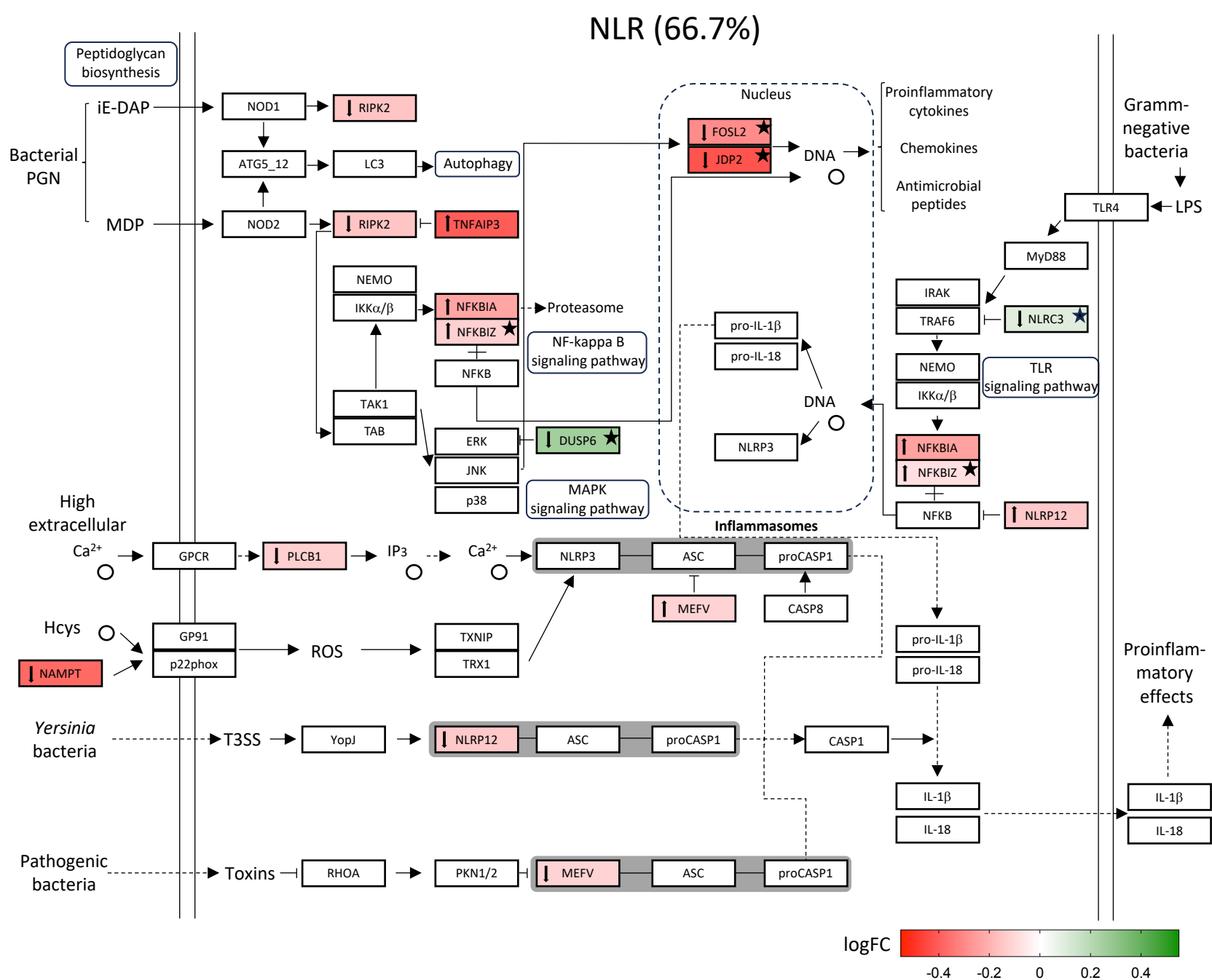

Supplement: S3 Fig — Representation of the pathways TLR (A) and NLR (B) following the design of KEGG. The given percentages reflect the number of in vivo vitamin D target genes, whose direction of regulation will contribute to pathway inhibition. Upregulated vitamin D targets are labeled green and downregulated red. Color intensity is proportional to logFC of gene expression between d1 and d0. Functionally similar proteins that are not indicated in KEGG are marked by an asterisk. (PDF) [file pone.0306426.s003.pdf]

A

## CHEMOKINE (50.0%)

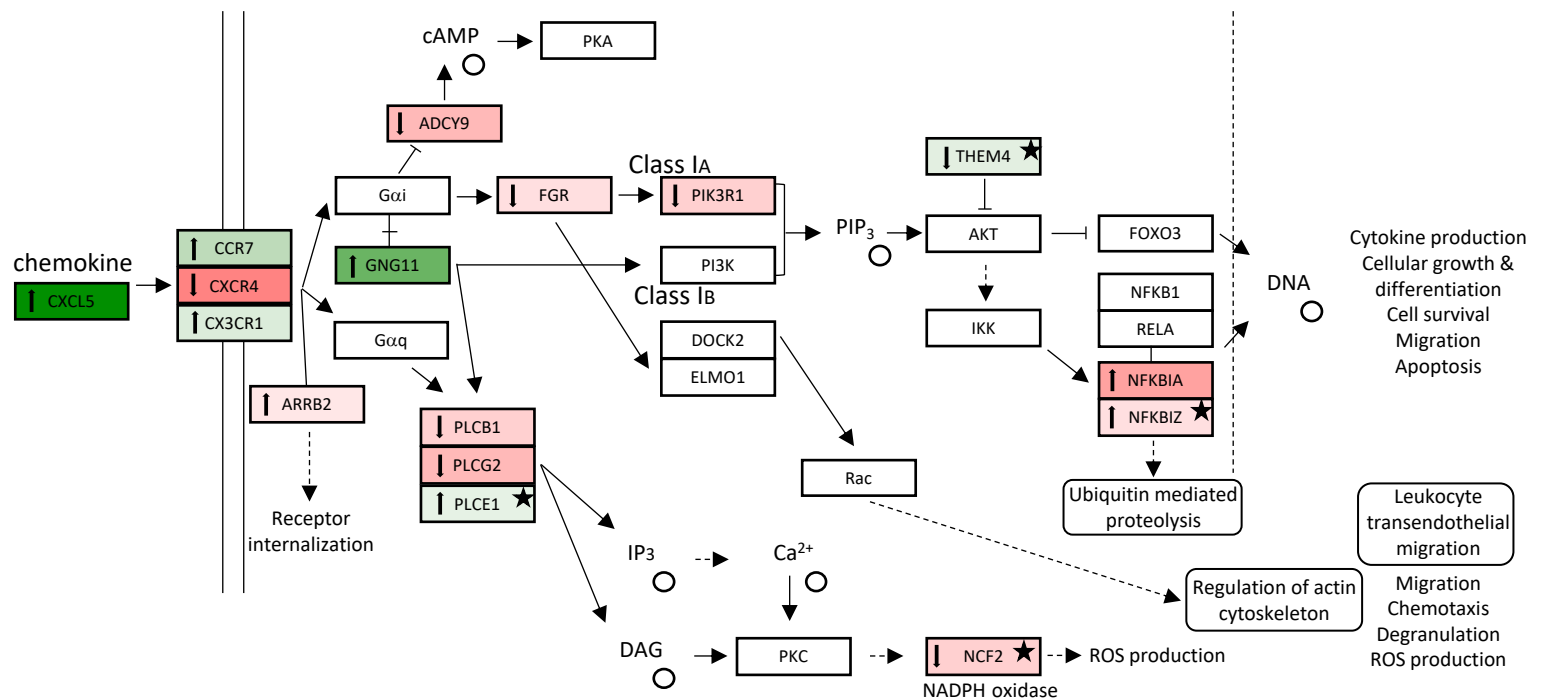

B

## IL17 (55.6%)

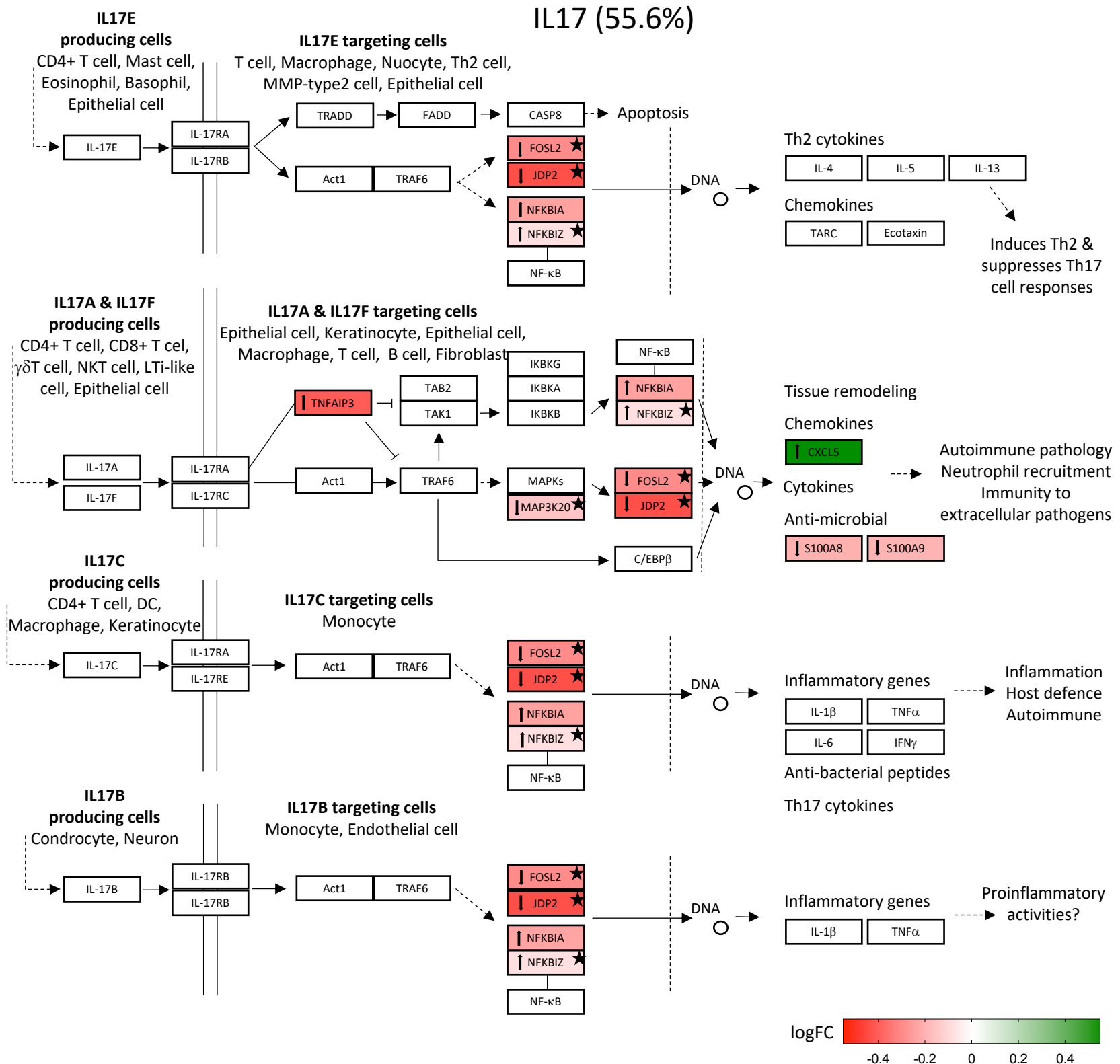

Supplement: S4 Fig — Representation of the pathways chemokine (A) and IL17 (B) signaling following the design of KEGG. The given percentages reflect the number of in vivo vitamin D target genes, whose direction of regulation will contribute to pathway inhibition. Upregulated vitamin D targets are labeled green and downregulated red. Color intensity is proportional to logFC of gene expression between d1 and d0. Functionally similar proteins that are not indicated in KEGG are marked by an asterisk. (PDF) [file pone.0306426.s004.pdf]

# PHAGOSOME (57.1%)

## Conventional phagocytosis

## ER-mediated phagocytosis

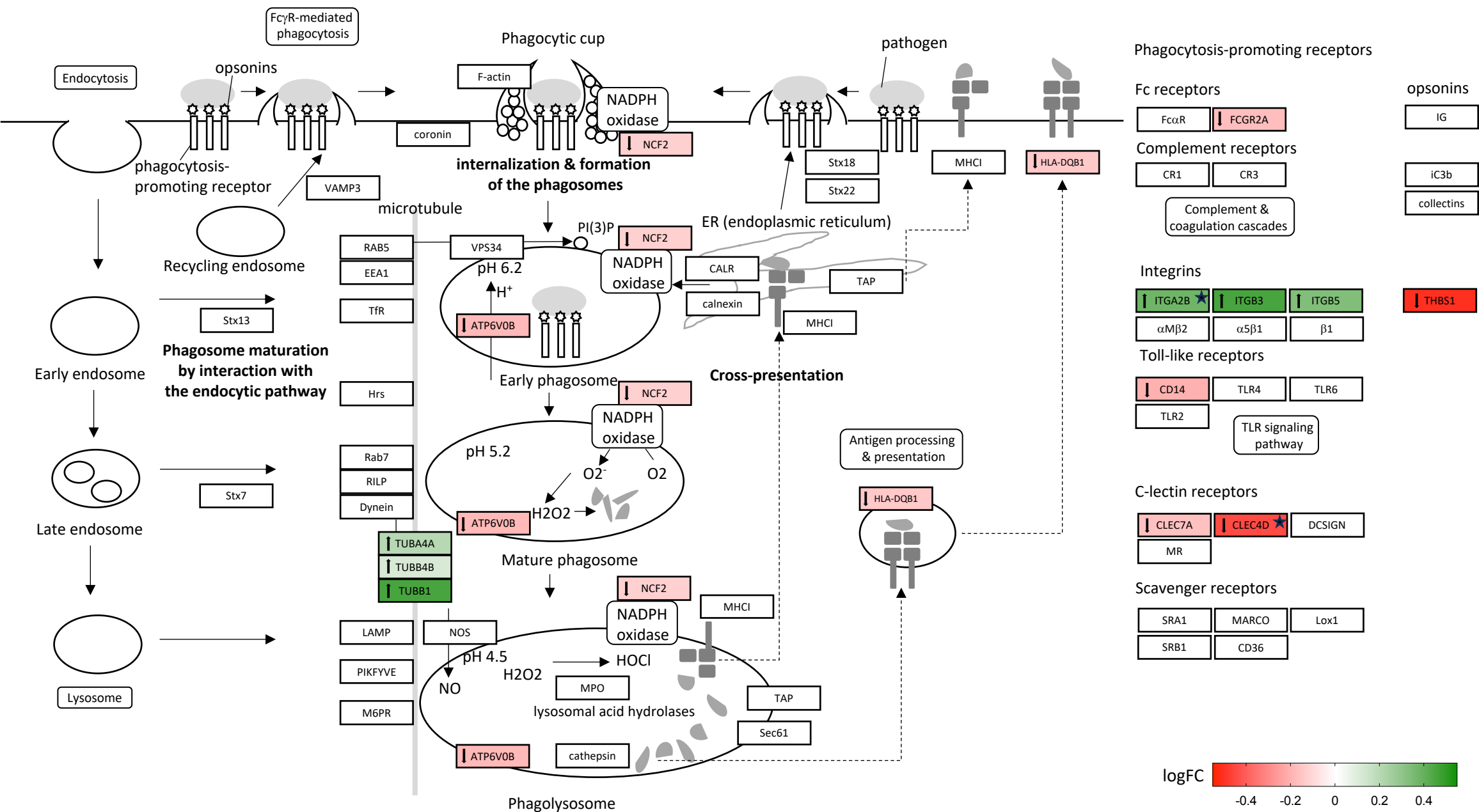

Supplement: S5 Fig — Representation of the phagosome pathway following the design of KEGG. The given percentages reflect the number of in vivo vitamin D target genes, whose direction of regulation will contribute to pathway inhibition. Upregulated vitamin D targets are labeled green and downregulated red. Color intensity is proportional to logFC of gene expression between d1 and d0. Functionally similar proteins that are not indicated in KEGG are marked by an asterisk. (PDF) [file pone.0306426.s005.pdf]

CLR (75.0%)

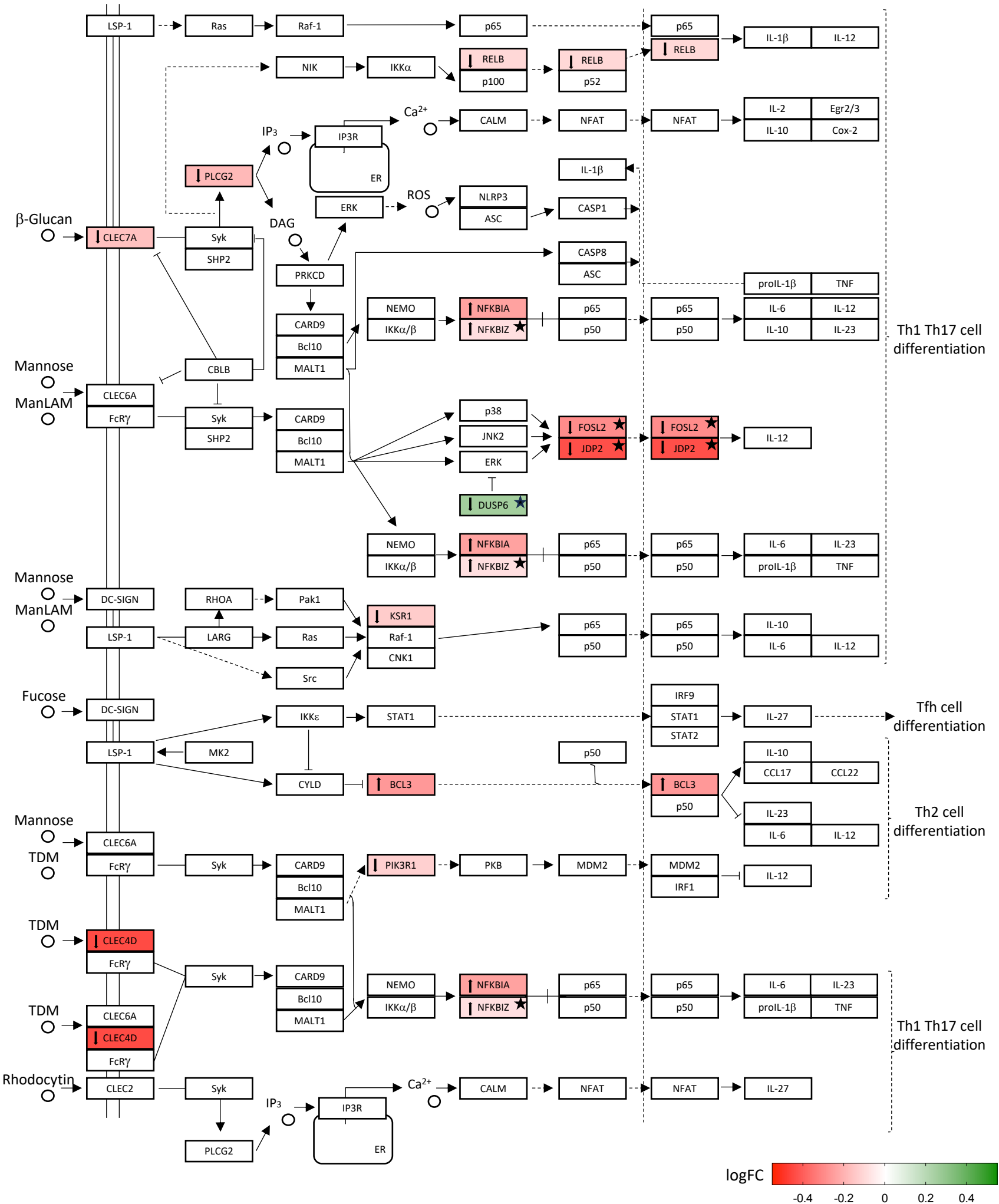

Supplement: S6 Fig — Representation of the CRL pathway following the design of KEGG. The given percentages reflect the number of in vivo vitamin D target genes, whose direction of regulation will contribute to pathway inhibition. Upregulated vitamin D targets are labeled green and downregulated red. Color intensity is proportional to logFC of gene expression between d1 and d0. Functionally similar proteins that are not indicated in KEGG are marked by an asterisk. (PDF) [file pone.0306426.s006.pdf]

# APOPTOSIS (55.0%)

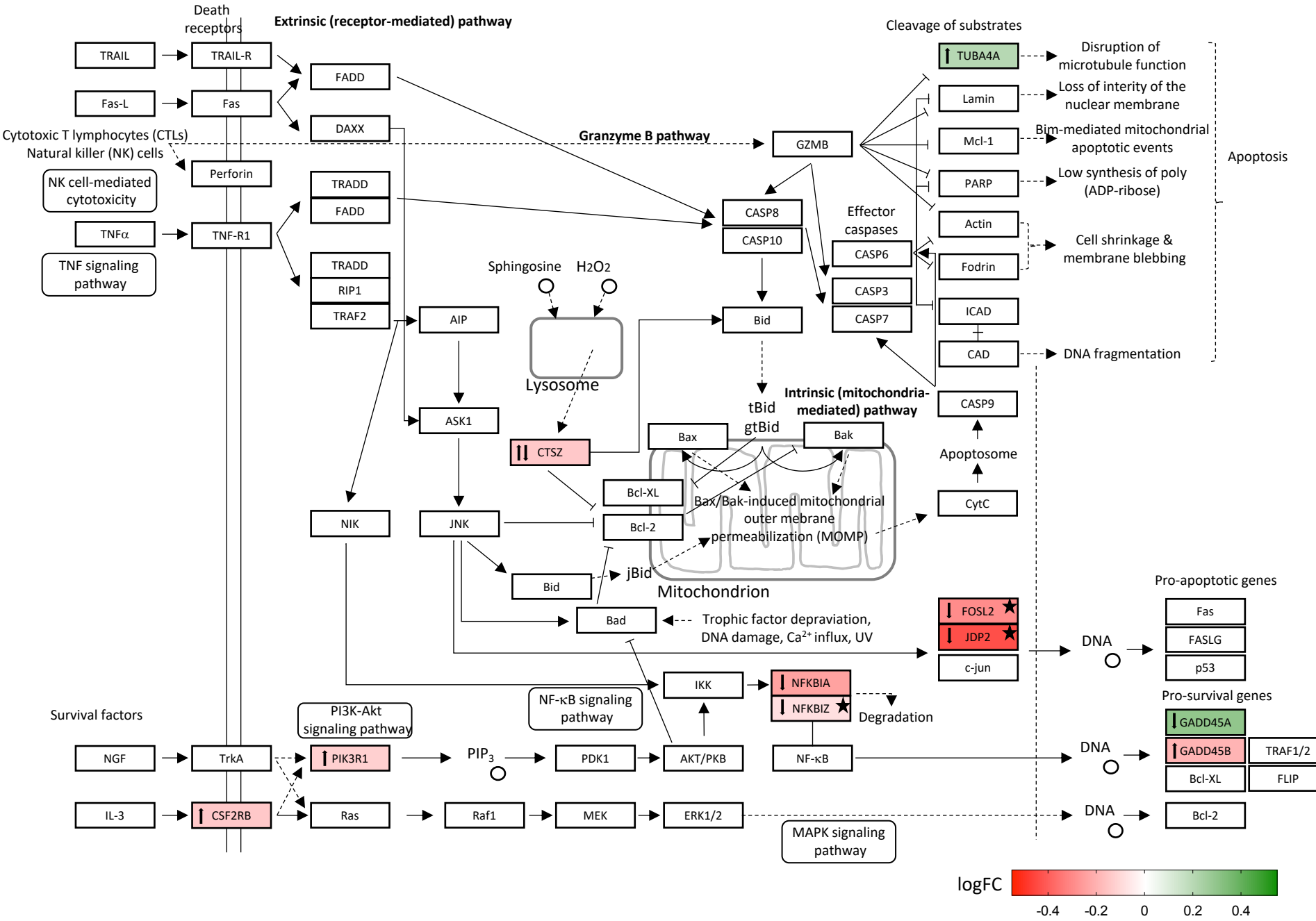

Supplement: S7 Fig — Representation of the apoptosis pathway following the design of KEGG. The given percentages reflect the number of in vivo vitamin D target genes, whose direction of regulation will contribute to pathway inhibition. Upregulated vitamin D targets are labeled green and downregulated red. Color intensity is proportional to logFC of gene expression between d1 and d0. Functionally similar proteins that are not indicated in KEGG are marked by an asterisk. (PDF) [file pone.0306426.s007.pdf]
