# Supplementary material for: In vivo vitamin D target genes interconnect key signaling pathways of innate immunity
Source: PLoS One. 2024 Jul 23;19(7):e0306426. doi: 10.1371/journal.pone.0306426 (PMC11265685; doi:10.1371/journal.pone.0306426)
Supplement: S8 Fig — The IGV browser was used to visualize ChIP-seq results for H3K4me3 (purple) [27], H3K27ac (green) [27] and VDR (red) [28] as well as FAIRE-seq data (turquois) [26] obtained in THP-1 cells that had been treated for 24 h with solvent (EtOH) or 1,25(OH)2D3 (1,25D). The target genes are classified based on strong (A) and weaker (B) VDR binding to their enhancers and TSS regions (shaded in grey). The peak tracks display merged data from the three biological repeats. Gene structures are shown in blue and vitamin D target genes are highlighted in red. The genomic regions 1 Mb up- and downstream of each gene’s TSS were inspected but only the areas relevant for 1,25(OH)2D3-dependent regulation are displayed. (ZIP) [file pone.0306426.s008.zip › S8B_Fig.pdf]

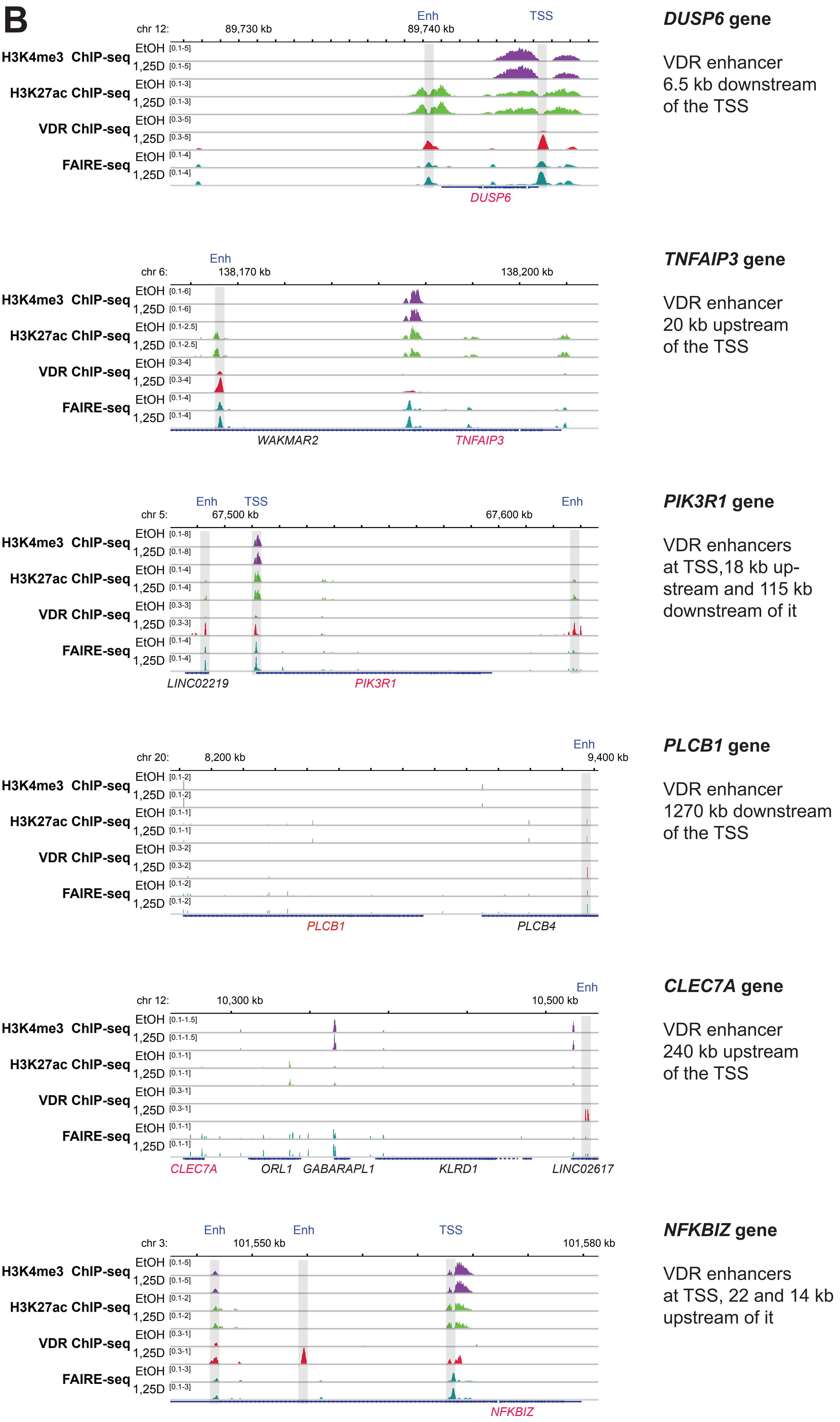
B
chr 12: 89,730 kb 89,740 kb
Enh TSS
H3K4me3 ChIP-seq
H3K27ac ChIP-seq
VDR ChIP-seq
FAIRE-seq
EtOH [0.1-5]
1,25D [0.1-5]
EtOH [0.1-3]
1,25D [0.1-3]
EtOH [0.3-5]
1,25D [0.3-5]
EtOH [0.1-4]
1,25D [0.1-4]
DUSP6
DUSP6 gene
VDR enhancer 6.5 kb downstream of the TSS
chr 6: 138,170 kb 138,200 kb
Enh
EtOH [0.1-6]
1,25D [0.1-6]
EtOH [0.1-2.5]
1,25D [0.1-2.5]
EtOH [0.3-4]
1,25D [0.3-4]
EtOH [0.1-4]
1,25D [0.1-4]
WAKMAR2
TNFAIP3
TNFAIP3 gene
VDR enhancer 20 kb upstream of the TSS
chr 5: 67,500 kb 67,600 kb
Enh TSS Enh
EtOH [0.1-8]
1,25D [0.1-8]
EtOH [0.1-4]
1,25D [0.1-4]
EtOH [0.3-3]
1,25D [0.3-3]
EtOH [0.1-4]
1,25D [0.1-4]
LINC02219
PIK3R1
PIK3R1 gene
VDR enhancers at TSS,18 kb upstream and 115 kb downstream of it
chr 20: 8,200 kb 9,400 kb
Enh
EtOH [0.1-2]
1,25D [0.1-2]
EtOH [0.1-1]
1,25D [0.1-1]
EtOH [0.3-2]
1,25D [0.3-2]
EtOH [0.1-2]
1,25D [0.1-2]
PLCB1
PLCB4
PLCB1 gene
VDR enhancer 1270 kb downstream of the TSS
chr 12: 10,300 kb 10,500 kb
Enh
EtOH [0.1-1.5]
1,25D [0.1-1.5]
EtOH [0.1-1]
1,25D [0.1-1]
EtOH [0.3-1]
1,25D [0.3-1]
EtOH [0.1-1]
1,25D [0.1-1]
CLEC7A
ORL1
GABARAPL1
KLRD1
LINC02617
CLEC7A gene
VDR enhancer 240 kb upstream of the TSS
chr 3: 101,550 kb 101,580 kb
Enh Enh TSS
EtOH [0.1-5]
1,25D [0.1-5]
EtOH [0.1-2]
1,25D [0.1-2]
EtOH [0.3-1]
1,25D [0.3-1]
EtOH [0.1-3]
1,25D [0.1-3]
NFKBIZ
NFKBIZ gene
VDR enhancers at TSS, 22 and 14 kb upstream of it
