# Supplementary material for: In vivo vitamin D target genes interconnect key signaling pathways of innate immunity
Source: PLoS One. 2024 Jul 23;19(7):e0306426. doi: 10.1371/journal.pone.0306426 (PMC11265685; doi:10.1371/journal.pone.0306426)
Supplement: S8 Fig — The IGV browser was used to visualize ChIP-seq results for H3K4me3 (purple) [27], H3K27ac (green) [27] and VDR (red) [28] as well as FAIRE-seq data (turquois) [26] obtained in THP-1 cells that had been treated for 24 h with solvent (EtOH) or 1,25(OH)2D3 (1,25D). The target genes are classified based on strong (A) and weaker (B) VDR binding to their enhancers and TSS regions (shaded in grey). The peak tracks display merged data from the three biological repeats. Gene structures are shown in blue and vitamin D target genes are highlighted in red. The genomic regions 1 Mb up- and downstream of each gene’s TSS were inspected but only the areas relevant for 1,25(OH)2D3-dependent regulation are displayed. (ZIP) [file pone.0306426.s008.zip › S8A_Fig.pdf]

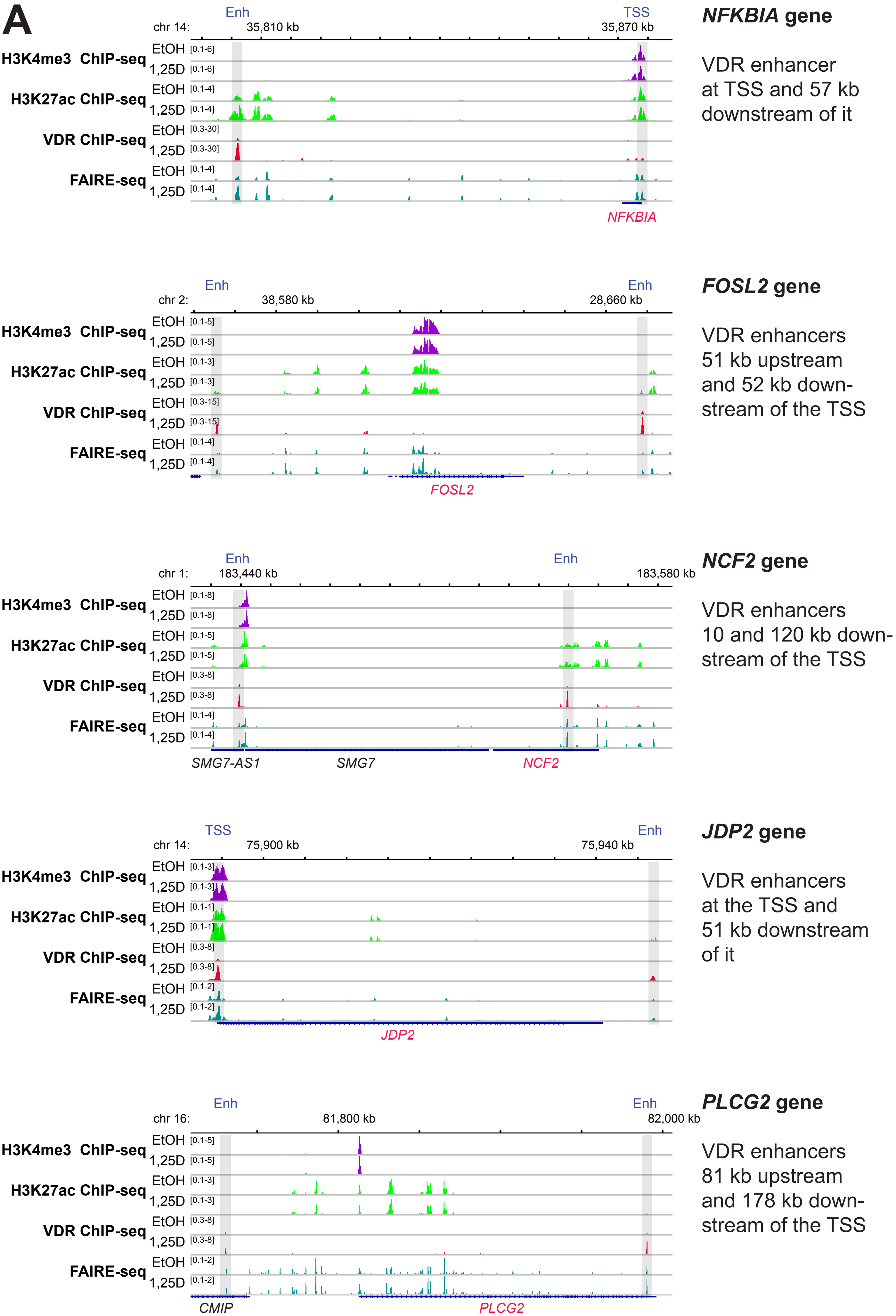
A
NFKBIA gene
VDR enhancer at TSS and 57 kb downstream of it
chr 14:
Enh
TSS
35,810 kb
35,870 kb
H3K4me3 ChIP-seq
H3K27ac ChIP-seq
VDR ChIP-seq
FAIRE-seq
EtOH
1,25D
[0.1-6]
[0.1-4]
[0.3-30]
NFKBIA
FOSL2 gene
VDR enhancers 51 kb upstream and 52 kb down-stream of the TSS
chr 2:
38,580 kb
28,660 kb
[0.1-5]
[0.1-3]
[0.3-15]
FOSL2
NCF2 gene
VDR enhancers 10 and 120 kb down-stream of the TSS
chr 1:
183,440 kb
183,580 kb
[0.1-8]
[0.3-8]
SMG7-AS1
SMG7
NCF2
JDP2 gene
VDR enhancers at the TSS and 51 kb downstream of it
chr 14:
75,900 kb
75,940 kb
[0.1-1]
[0.1-2]
JDP2
PLCG2 gene
VDR enhancers 81 kb upstream and 178 kb down-stream of the TSS
chr 16:
81,800 kb
82,000 kb
CMIP
PLCG2
